# Supplementary material for: Urinary Continence Resolution after Bariatric Surgery: Long-Term Results after Six-Year Follow-Up
Source: J Clin Med. 2023 Mar 8;12(6):2109. doi: 10.3390/jcm12062109 (PMC10051985; doi:10.3390/jcm12062109)
Supplement: Supplementary file 1 [file jcm-12-02109-s001.zip › jcm-2173904-SI.pdf]

Questionnaire international sur l'incontinence = ICIQ

Beaucoup de gens ont des fuites d'urine. Nous essayons de savoir combien de personnes présentent ces problèmes et quelle gène ils représentent. Nous vous remercions de bien vouloir répondre aux questions ci-dessous, en sachant que les questions se rapportent à la moyenne, au cours du dernier mois.

1. A quelle fréquence avez-vous des pertes d'urine ?

Jamais	0
Environ une fois par semaine, ou moins	1
Deux à trois par semaine	2
Environ une fois par jour	3
Plusieurs fois par jour	4
Toujours	5

2. Nous voudrions savoir quelle quantité vous pensez fuir. Combien vous fuyiez en général (que vous portiez des garnitures ou non)

Pas de fuites	0
Un petit peu	2
Une quantité moyenne	4
Une large quantité	6

3. En moyenne, dans quelle mesure vos fuites retentissent sur votre vie quotidienne ? Entouré un nombre de 0 (pas du tout) à 10 (considérablement)

0 1 2 3 4 5 6 7 8 9 10

Pas du tout *considérable*

Score ICI-Q : score totale 1+2+3 =
